# Clinical Response to the CD95-Ligand Inhibitor Asunercept Is Defined by a Pro-Inflammatory Serum Cytokine Profile

**DOI:** 10.3390/cancers12123683

**Published:** 2020-12-08

**Authors:** Aleksandar Radujkovic, Tobias Boch, Florian Nolte, Daniel Nowak, Claudia Kunz, Alexandra Gieffers, Carsten Müller-Tidow, Peter Dreger, Wolf-Karsten Hofmann, Thomas Luft

**Affiliations:** 1 Department of Internal Medicine V, University Hospital Heidelberg, 69120 Heidelberg, Germany; carsten.mueller-tidow@med.uni-heidelberg.de (C.M.-T.); peter.dreger@med.uni-heidelberg.de (P.D.); thomas.luft@med.uni-heidelberg.de (T.L.); 2 Department of Hematology and Oncology, University Hospital Mannheim, Heidelberg University, 68167 Mannheim, Germany; tobias.boch@medma.uni-heidelberg.de (T.B.); florian.nolte@medma.uni-heidelberg.de (F.N.); daniel.nowak@medma.uni-heidelberg.de (D.N.); w.k.hofmann@medma.uni-heidelberg.de (W.-K.H.); 3 Apogenix AG, 69120 Heidelberg, Germany; claudiakunz@gmx.de (C.K.); a.gieffers@web.de (A.G.)

**Keywords:** asunercept, APG101, anemia, myelodysplastic syndrome, MDS

## Abstract

**Simple Summary:**

Asunercept showed promising clinical efficacy in anemic, transfusion-dependent patients with low and intermediate risk myelodysplastic syndrome. In this retrospective post hoc analysis, serum levels of biomarkers were measured in study patients focusing on cytokines associated with erythropoiesis, inflammation, apoptosis, bone marrow fibrosis, and inflammasome activity. Baseline serum biomarkers were correlated with treatment response in order to propose a hypothetical responder serum profile. Response to asunercept was associated with improved overall survival. Higher baseline values of interleukin-18 (IL-18), S100 calcium-binding protein A9 (S100A9) and soluble p53 were predictive of non-response to asunercept. Non-responding patients showed a distinct, pro-inflammatory serum cytokine profile which was persistent throughout the first half of the treatment phase and appeared unaffected by asunercept. Our post hoc analysis suggests that serum cytokine profiling based on IL-18, S100A9 and soluble p53 may represent an approach to identify and select low-risk myelodysplastic syndrome patients most likely to benefit from asunercept treatment.

**Abstract:**

Asunercept (APG101) is a well-tolerated CD95-ligand inhibitor that showed promising efficacy in a prospective, single-arm phase I study in anemic, transfusion-dependent patients with low and intermediate risk myelodysplastic syndrome (MDS). In this retrospective post hoc analysis, serum levels of biomarkers were measured in study patients focusing on cytokines associated with erythropoiesis, inflammation, apoptosis, bone marrow fibrosis, and inflammasome activity. Baseline serum biomarkers were correlated with treatment response, in order to propose a hypothetical responder serum profile. After an updated median follow-up of 54 months (range 7–65), response to asunercept was associated with improved overall survival (at 3-years: 67% [95%CI 36–97] versus 13% [95%CI 0–36] in responders versus non-responders, respectively). Higher baseline values of interleukin-18 (IL-18), S100 calcium-binding protein A9 (S100A9) and soluble p53 were predictive of non-response to asunercept (area under the receiver operating characteristic curve 0.79–0.82). Furthermore, non-responding patients showed a distinct, pro-inflammatory serum cytokine profile which was persistent throughout the first half of the treatment phase and appeared unaffected by asunercept. Although prospective validation is required, our post hoc analysis suggests that serum cytokine profiling based on IL-18, S100A9 and soluble p53 may represent an approach to identify and select low-risk MDS patients most likely to benefit from asunercept treatment.

## 1. Introduction

Myelodysplastic syndromes (MDS) are a heterogeneous group of clonal disorders characterized by dysplastic bone marrow (BM) morphology, inefficient hematopoiesis and a variable risk of leukemic transformation. Importantly, anemia is the most frequent clinical finding in MDS patients [1].

For erythropoiesis-stimulating agents (ESA), response rates range from 45% to 73% and from 25% to 75% for ESA-naïve and previously treated patients, respectively [2]. Importantly, the duration of response is limited, i.e., all patients ultimately develop ESA resistance becoming transfusion-dependent again. Luspatercept, an activin receptor type IIB fusion ligand trap novel agent, was recently approved to reduce the severity of anemia in patients with lower-risk MDS with ring sideroblasts who failed or were unlikely to respond to ESA [3,4]. However, ring sideroblast positive patients only represent a minor fraction of MDS patients with the result that blood transfusion often remains the only resort for anemic patients with lower risk MDS.

In addition to compromised quality of life and complications of infection and iron overload, transfusion-dependent anemia is associated with significant costs for healthcare systems [5,6]. Consequently, in view of an aging population and the increasing number of MDS patients in the future, there is an unmet medical need for efficient treatment of anemic patients with lower risk MDS.

The CD95 signaling pathway is one of the negative regulators of erythropoiesis [7]. In particular, immature CD95-positive erythroid progenitors undergo apoptosis when interacting with its ligand (CD95L) expressed on the more mature erythroblasts [8]. Indeed, overexpression of CD95L and inadequate activation of the CD95 system which results in a pro-apoptotic BM milieu has been demonstrated in MDS and appears to contribute to the anemia observed in patients with low-risk diseases [9,10].

Asunercept (APG101) is a recombinant fusion protein that inhibits CD95 activation via binding to CD95L expressed on target cells and active CD95L in solution. In vitro, asunercept was demonstrated to rescue erythropoiesis in MDS stem cells independent of the expression level of CD95 or CD95L [11]. Recently, asunercept has been evaluated in an open-label, single arm phase I clinical trial, including 20 transfusion-dependent patients with low to intermediate-1 risk MDS who were refractory to ESA (EudraCT No 2012-003027-37). In sum, asunercept was overall well tolerated and showed promising efficacy as reflected by a decrease in transfusion requirement in 9 out of the 20 enrolled study patients [12].

In this retrospective post hoc study, we evaluated serum levels of cytokines associated with erythropoiesis (activin A, follistatin, bone morphogenetic protein 7 [BMP7]) [13,14], apoptosis and bone marrow fibrosis (receptor activator of NF-κB [RANK] ligand, soluble p53) [15,16], inflammation (interleukin-18 [IL-18], interferon gamma [IFNγ], chemokine (C-X-C motif) ligand 9 [CXCL9]) and inflammasome activation (S100 calcium-binding protein A9 [S100A9], interleukin-1 beta [IL-1β], interleukin-37 [IL-37], high-mobility-group-protein B1 [HMGB1]) [17,18] in patients enrolled in the aforementioned clinical trial. The results were correlated with treatment response (i.e., reduction of transfusion requirements), in order to propose a hypothetical asunercept responder serum profile.

## 2. Results

### 2.1. Patient Characteristics and Drug Safety

A total of 20 transfusion-dependent patients were enrolled in the study and received at least one dose of asunercept (intent-to-treat population, for further details see Boch et al. [12]). The results regarding primary and secondary study endpoints including safety were published previously [12]. One patient withdrew consent for blood sampling and for monitoring of follow-up and was therefore excluded from the present analysis. Patient, disease and treatment characteristics of the remaining patients (n = 19) are summarized in Table 1.

### 2.2. Response to Asunercept Treatment

**Figure 1:**
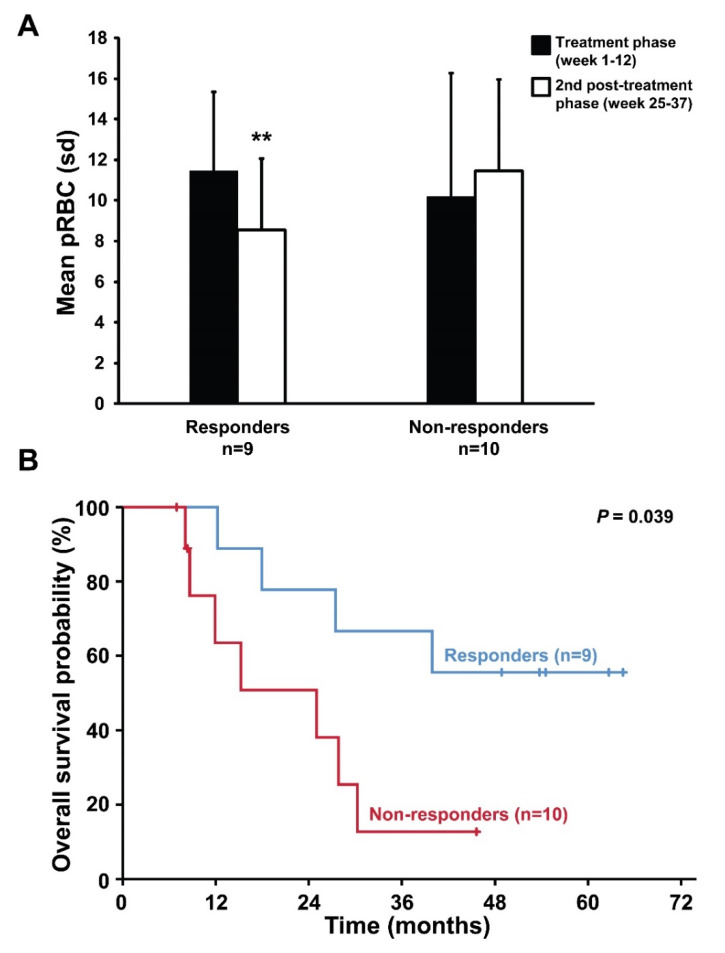
Transfusion requirement and survival analysis according to response to treatment with asunercept. (**A**) Mean number of transfused packed red blood cells (pRBC) during the treatment phase (week 1–12) and second half of the post-treatment phase (week 25–37) in responding versus non-responding patients (sd, standard deviation; ** *p* < 0.01). (**B**) In retrospective post hoc analysis, response to asunercept was associated with improved overall survival (OS, logrank *p* = 0.039). After a median follow-up of 54 months (range 7–65), OS at 3 years was 67% (95% CI 36–97) in responding patients versus 13% (95% CI 0–36) in non-responding patients.

Briefly, and as previously reported in detail [12], the number of transfused packed red blood cells (pRBC) developed from a mean of 10.8 (±5.1) during the treatment phase over a mean pRBC of 9.9 (±4.9) during the first 12-week follow-up period, and to a mean of 10.0 (±4.2) during the second 12-week follow-up period. Further analysis revealed that the overall decline of the transfusion requirement following asunercept treatment could be attributed to a subgroup of 9 patients in whom transfusion requirement decreased towards the end of study (Figure 1A, see also Boch et al. [12]).

Except for median serum C-reactive protein (CRP) level at baseline which was slightly higher in non-responding patients (6.1 versus 2.6 mg/L, *p* = 0.026), no differences in disease and treatment characteristics between “responders” (n = 9) and “non-responders” (n = 10) were observed (Table 1).

### 2.3. Follow-up and Survival According to Treatment Response

Median follow-up at the time of the retrospective analysis was 54 months (range 7–65). A total of 11 patients had died by the time of analysis. In one patient, progression to acute myeloid leukemia was documented. Of the 11 deceased patients, cause of death was documented as fatal infection in 5, “frailty” in 2, and fatal accident in 1 patient. Fatal infection due to neutropenia was documented in 3 patients. The cause of death was unknown for 3 patients.

In responding patients, the cause of death was documented as “frailty” in 1 patient, fatal accident in 1 patient and as unknown in 2 patients. In non-responders, the cause of death was documented as fatal infection in 5 patients (due to neutropenia in 3 patients), “frailty” in 1 patient and as unknown in 1 patient. Retrospectively, response to asunercept was associated with improved overall survival (OS) (Figure 1B).

Information on the mutational status of genes at baseline was available for *TP53*, *ASXL1*, *EZH2*, *ETV6* and *RUNX1*. None of the study patients carried mutations in *TP53*, *EZH2* or *ETV5*. Presence of mutations in *ASXL1* or *RUNX1* was not correlated with response to asunercept.

### 2.4. Biomarker Serum Levels and Response to Treatment with Asunercept

Baseline biomarker serum levels according to treatment response are given in Table 2. In responding patients, median baseline serum levels of IL-18, S100A9 and soluble p53 were lower (i.e., approximately 1.7-, 1.4- and 66-fold, respectively, as compared to non-responders). In addition, there was a trend towards lower baseline follistatin levels in responders.

Baseline IL-18 levels were positively correlated with IFNγ, CXCL9 and follistatin, whereas no correlation of IL-18 with markers of inflammasome activity was observed. Activin was inversely correlated with follistatin and baseline IL-18. A high level of inter-correlation was observed between the markers of inflammasome activity S100A9, IL-1β and IL-37. Baseline levels of RANK ligand and soluble 53 were positively correlated with each other and were both positively correlated with baseline IL-1 β, HMGB1 and IL-37. The corresponding correlation matrix is given in Table 3.

Higher baseline serum levels of IL-18 and soluble p53 levels were associated with increased odds of treatment non-response (Table 4). For baseline S100A9, only a trend in this regard was found. As per area under the ROC curve analysis IL-18, S100A9 and soluble p53 were predictive of non-response to asunercept, with IL-18 being the most informative cytokine in this regard (Table 4). In contrast, no associations of other investigated biomarkers with treatment response were observed (Table 4).

**Figure 2:**
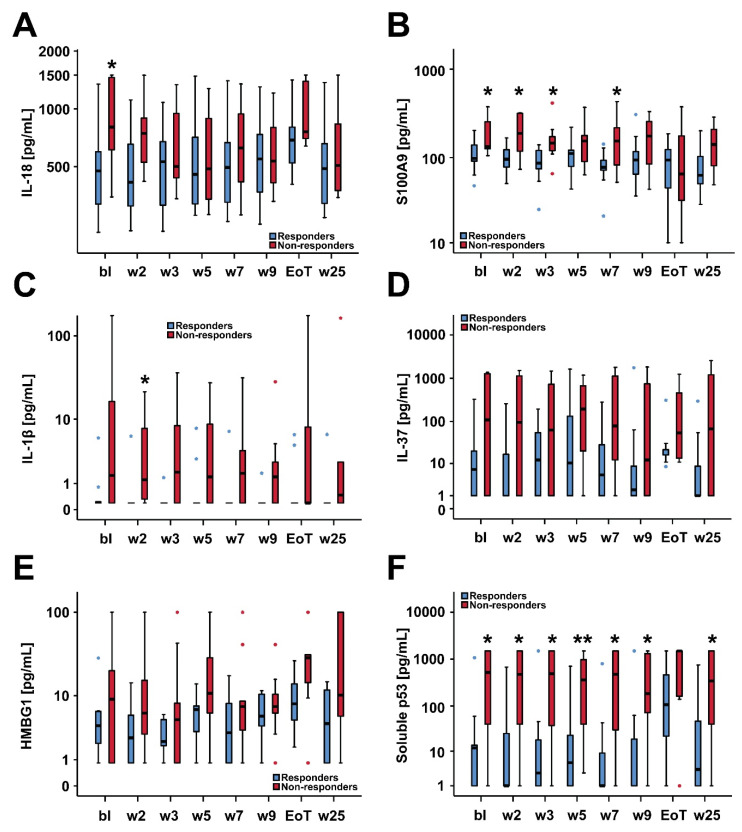
Box plot diagrams of serum levels of IL-18 (**A**), S100A9 (**B**), IL-1β (**C**), IL-37 (**D**), HMGB1 (**E**) and soluble p53 (**F**) in responding (n = 9) versus non-responding (n = 10) patients at defined study visits. Median serum biomarker levels were compared in responding versus non-responding patients applying the Mann–Whitney test (* *p* < 0.05, ** *p* < 0.01). Abbreviations: bl, baseline; w, week; EoT, end of treatment (i.e., week 13 visit).

**Figure 3:**
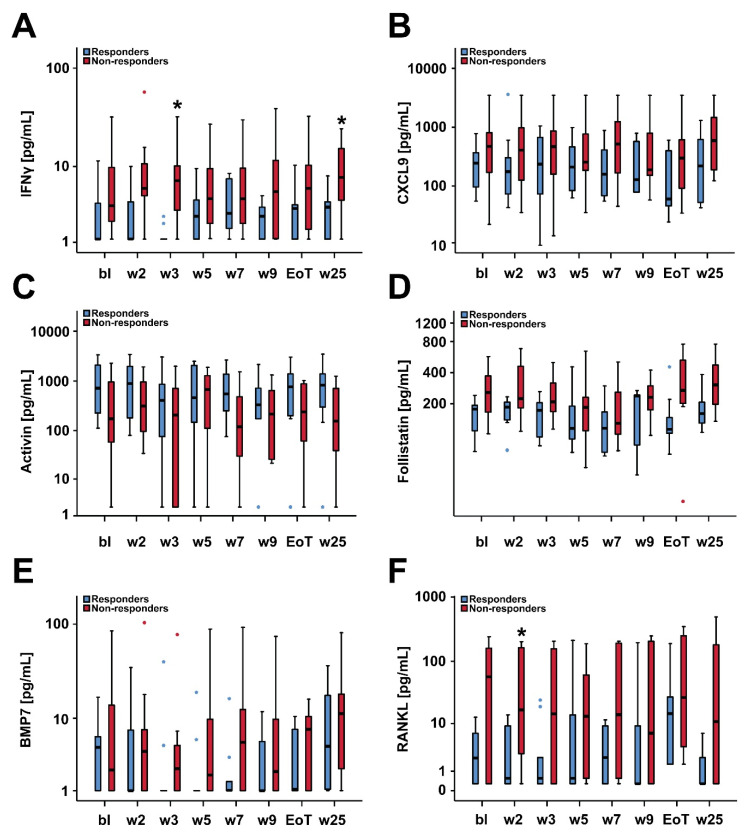
Box plot diagrams of serum levels of IFNγ (**A**), CXCL9 (**B**), activin (**C**), follistatin (**D**), BMP7 (**E**) and RANKL (**F**) in responding (n = 9) versus non-responding (n = 10) patients at defined study visits. Median serum biomarker levels were compared in responding versus non-responding patients applying the Mann–Whitney test (* *p* < 0.05). Abbreviations: bl, baseline; w, week; EoT, end of treatment (i.e., week 13 visit).

When regarding serum biomarker levels assessed at different pre-defined study time-points (Figure 2 and Figure 3), non-responding patients showed a distinct, rather pro-inflammatory serum cytokine profile which appeared to be persistent throughout the first half of the treatment phase, in particular and significantly with respect to S100A9 and soluble p53 (Figure 2B,F). This trend towards a persistent systemic pro-inflammatory serum profile appeared to be rather unaffected by asunercept.

### 2.5. Survival and Baseline Serum Levels of IL-18, S100A9 and Soluble p53

Finally, associations of baseline serum levels of IL-18, S100A9 and soluble p53 with survival probability were analyzed. In the entire cohort, higher levels of baseline IL-18 correlated with worse OS (hazard ratio [HR] 2.43 per doubling in baseline IL-18 level, 95% CI 1.11–5.30, *p* = 0.026), whereas no significant associations for S100A9 and soluble p53 were observed (HR 1.78 [95% CI 0.81–3.90], *p* = 0.148 and HR 1.06 [95% CI 0.91–1.23], *p* = 0.447, respectively).

## 3. Discussion

The CD95L inhibitor asunercept has shown a positive safety profile and promising efficacy in the phase I clinical trial in heavily transfusion-dependent patients diagnosed with lower risk MDS [12]. The potential clinical efficacy of asunercept was based on a reduction of transfused pRBC during the course of the study, which, however, was attributable to a subgroup of nine patients (“responders”). Interestingly, most of the “responders” showed a continuous reduction of their transfusion requirement during the second half of the 24-week follow-up period after the end of the treatment phase, indicating rather slow dynamics of the asunercept effect, which may result in delayed clinical efficacy. However, non-responding patients showed a distinct, rather pro-inflammatory serum cytokine profile, which was persistent throughout the first half of the treatment phase and appeared rather unaffected by asunercept. With longer follow-up than in the previous report [12], response to asunercept was associated with prolonged survival as suggested by the present post hoc analysis of the study cohort.

There is strong evidence pointing to a critical role of the BM microenvironment in the pathobiology of MDS [20,21]. Alterations and abnormalities of the BM microenvironment involving aberrant cytokine expression or cytokine imbalance, inflammatory changes and immune dysfunction are thought to precede and facilitate clonal evolution in MDS contributing to disease phenotype and outcome [21]. MDS patients were shown to exhibit an abnormal cytokine milieu, probably derived from the interaction of stromal cells with the MDS clone in the BM, resulting in immune dysregulation and a persistent pro-inflammatory state [22,23].

Of the analyzed serum factors, baseline levels of S100A9, IL-18, and soluble p53 were significantly associated with clinical response to asunercept treatment. Recently, activation of the NLRP3 inflammasome, which results in caspase-1-dependent pyroptotic cell death and generation of generation of IL-1β and IL-18 in their active forms, was shown to suppress normal hematopoiesis and promote clonal expansion observed in patients with low-risk MDS [18,24]. In particular, the alarmin S100A9, the levels of which were shown to be elevated in low-risk MDS [25,26], appears to play a pivotal role in triggering NLRP3 inflammasome activation and propagation of the dysplastic clone [24,27]. In addition, both S100A9 and IL-18 were shown to drive accumulation and activation of myeloid-derived suppressor cells (MDSCs) that promote immune suppression and tolerance, contributing to the biological phenotype of clonal evolution and ineffective hematopoiesis in MDS [24,27,28].

Notably, in myeloid cells, CD95L was also reported to induce the release of biologically active IL-18 independent of inflammasome activation [29,30]. Since overexpression of CD95 constitutes a hallmark of ESA resistance [11], the elevated levels of IL-18 observed in non-responders in our study might reflect an enhanced activity of the CD95 signaling pathway, thereby facilitating resistance or non-response to asunercept, particularly in an otherwise pro-inflammatory environment. The observation that an inflammatory state compromises therapy of anemia in MDS patients is not new and could be also demonstrated for tumor necrosis factor-alpha and IL-1β in the context of ESA treatment [31,32].

Data on prognostic relevance of soluble p53 are scarce. With regard to hematologic malignancies, soluble p53 has been investigated in studies on Hodgkin’s disease [33] and chronic lymphocytic leukemia (CLL) [34], and in one study, on MDS [16]. In lymphoma patients, elevated levels of soluble p53 were associated with treatment failure and poor survival [33,34]. Interestingly, in the CLL study [34], and similar to our observations, no particular variations in the level of soluble p53 were noted during the course of follow-up, indicating the putative stability of this marker. However, for MDS, no correlations with clinical parameters and outcome were apparent [16], and the present study is, to our knowledge, the first to suggest a prognostic relevance for low-risk MDS in the context of asunercept therapy. Certainly, with respect to the observations in CLL [34], elevated levels of soluble p53 may solely be the result of a higher cell turnover rate due to a more aggressive disease biology. However, given the involvement of inflammasomes in DNA damage responses induced by cellular stress [35], persistently elevated levels of soluble p53 might also reflect enhanced inflammatory or otherwise immunogenic programmed cell death rates in low-risk MDS patients not responding to asunercept in our series.

In light of these considerations and of our results, one might speculate that response to asunercept is affected by a systemic pro-inflammatory state involving high levels of S100A9 and IL-18 and, as a consequence, high soluble p53. In absence of direct experimental evidence, this pro-inflammatory serum cytokine profile may be related to an enhanced inflammasome activity that impairs asunercept effects on erythropoiesis. It should, however, be pointed out that for other factors involved in or produced by inflammasome activation (IL-1β, HMGB1, IL-37), no correlations with treatment response were observed, possibly owing to the small sample size. Further, it should be noted that distinct (pro-inflammatory) cytokine signatures associated with prognosis or response to treatment may solely reflect the host response to a more aggressive disease state that is not necessarily captured by common disease risk scoring systems.

It should be noted that, in the present study, which included low and intermediate risk patients with MDS, the proportion of fatal infections was lower in patients responding to asunercept. With regard to immunologic effects, asunercept was found to prevent the development of graft-versus-host disease, while preserving graft-versus-leukemia effects in murine transplant models [36]. Consequently, treatment with asunercept probably involves immunosuppressive, or rather immunomodulating effects. However, although baseline disease characteristics (including neutrophil counts) did not differ between responders and non-responders, the higher proportion of fatal infections in non-responding patients is likely to be attributed to progressive MDS after the end of the treatment phase involving both refractory anemia and progressive neutropenia.

The main limitations of our study are represented by the limited number of patients and the observational and retrospective study design. The results should, therefore, be regarded with this caveat in mind. Certainly, further validation of our results is required, and currently, preparations are ongoing for a larger multicenter phase II proof-of-concept trial, in order to evaluate the safety and efficacy of asunercept in combination with ESA in patients with low-risk MDS. In addition to a laboratory study on circulating cytokine levels covering the response biomarkers suggested by the present study, the follow-up study will comprise further in-depth analyses of primary patient cells (including MDSCs) on the transcriptome and proteome level, in order to elucidate pathways linking inflammasome activation with treatment response to asunercept.

## 4. Materials and Methods

### 4.1. Study Design, Study Population, Treatment Schedule and Objectives

Study details were published recently [12]. Briefly, asunercept was investigated in a prospective, open-label, single arm phase I study (EudraCT No 2012-003027-37; NCT01736436), which was performed in accordance with ICH-GCP guidelines. Included patients had confirmed diagnosis of MDS with low or intermediate risk according to WPSS [37]. Enrolled patients were all transfusion-dependent (at least 4 units or pRBC during the last 8 weeks prior to study inclusion) and were refractory to ESA (as assessed after at least 8 weeks of treatment with ESA) or had low probability to respond to ESA treatment according to the Nordic group [38]. Written informed consent according to the Declaration of Helsinki was obtained for all patients, and the ethics committee had approved the sample and data collection (Reference number: 2012-058F-MA).

The study consisted of a 4-week screening period, followed by a 12-week treatment period in which asunercept was given intravenously once weekly at two dose levels (100 mg and 400 mg). After completion of the treatment phase (end of treatment, EoT = week 13), patients entered a 24-week follow-up period (divided into a first and second 12-week follow-up phase) until the end-of-study visit (EoS = week 37).

### 4.2. Collection of Serum Samples and Biomarker Assessments

Patient blood specimens were collected at the following visits: pre-dose at baseline (i.e., before first infusion of asunercept), pre-dose at week 2, 3, 5, 7 and 9 (before the respective asunercept infusion), at week 13 (=EoT), and at week 25 (after completion of the first 12-week post-treatment period). Serum was prepared from blood samples by centrifugation and stored immediately at (or below) −80 °C.

Serum levels of cytokines associated with erythropoiesis (activin A, follistatin, BMP7), apoptosis and BM fibrosis (RANK ligand, soluble p53), inflammation (IL-18, IFNγ, CXCL9) and inflammasome activity (S100A9, IL-1β, IL-37, HMGB1) were measured retrospectively using commercial ELISA Kits according to the manufacturers’ instructions. Except for follistatin (Human Follistatin Construction Kit from Antigenix, Huntington Station, NY, USA), R&D Duoset Kits (R&D Systems Europe Ltd., Abingdon, UK) were applied.

Molar ratio of follistatin:activin was calculated by converting pg/mL to pmol/L (by dividing by the molecular weight of each protein) and then expressed as a ratio.

### 4.3. Statistical Analysis

Categorical and continuous variables of patient characteristics were compared using Fisher’s exact test and the Mann–Whitney test, respectively. The comparison of the transfusion burden (treatment phase [week 1–12] versus second post-treatment phase [week 25–37]) was performed by using a matched-pairs Wilcoxon signed rank test.

Cytokine data were deemed to be non-parametric according to the Shapiro–Wilk test (*p* < 0.05) and were described using the median (with corresponding interquartile ranges [IQR]) and were compared between responding and non-responding, applying the Mann–Whitney test. The inter-relationship between baseline cytokine serum levels was examined using Spearman’s correlation coefficient. To control for multiple testing, false discovery rates were calculated following the Benjamini–Hochberg procedure [19].

Biomarker serum levels at baseline were correlated with treatment response post hoc in order to define a hypothetical responder serum profile. The performance of each serum marker at baseline in identifying non-response to asunercept was assessed by receiver operating characteristic (ROC) curve analysis with calculation of the area under the curve (AUC). In addition, univariate logistic regression analyses of baseline serum levels with the endpoint non-response were performed. In absence of established reference ranges, cytokine concentrations were analyzed as continuous variables. Since all cytokine serum levels showed skewed distributions, data were log2 transformed. Consequently, odds ratios (OR) from these models refer to the increase in odds of non-response for a twofold increase in the corresponding baseline cytokine level.

Distributions of survival times were estimated by the method of Kaplan and Meier. The confidence interval (CI) estimation was performed using Greenwood’s formula for the variance of the survival function. The follow-up times were calculated by the reverse Kaplan–Meier estimate [39]. Overall survival (OS) was calculated from the date of the first dose of study drug to death of any cause. Patients alive were censored at the date of last contact. Comparison of OS was done using the log rank test. For the univariable analysis of the associations between selected baseline cytokine levels (as continuous variables) and OS, Cox regression models were applied.

Calculations were done using IBM^®^ SPSS^®^ Statistics, Version 25.0 (IBM, Armonk, USA). All statistical tests were two-sided. Effects were estimated with 95% confidence interval (95% CI). Results with *p* values < 0.05 were considered to be statistically significant.

## 5. Conclusions

Our results demonstrate the presence of a pro-inflammatory systemic environment in patients not responding to asunercept. Although prospective validation is required, serum cytokine profiling including IL-18, S100A9 and soluble p53 and probably other factors associated with inflammasome activation may represent an approach to identify and select anemic patients with lower risk MDS most likely to benefit from asunercept treatment.

## Figures and Tables

**Table 1:** Patient characteristics according to treatment response.

	Entire Cohort n = 19	Non-Responders n = 10	Responders n = 9	*p*
Median age (years, range)	75 (56–82)	75 (56–77)	76 (62–82)	0.901
Age category, n (%)				0.582
<65 years	3 (16)	1 (10)	2 (22)	
≥65 years	16 (84)	10 (90)	7 (78)	
Sex, n (%)				0.999
Male	12 (63)	6 (60)	6 (67)	
Female	7 (37)	4 (40)	3 (33)	
ECOG at baseline, n (%)				0.482
0	7 (41)	2 (25)	5 (56)	
1	6 (35)	3 (38)	3 (33)	
2	3 (18)	2 (25)	1 (11)	
3	1 (6)	1 (13)	0 (0)	
Missing	2	2		
WHO subtype, n (%)				0.211
RARS	1 (5)	0 (0)	1 (11)	
RCMD	16 (84)	9 (90)	7 (78)	
RCMD-RS	1 (5)	1 (10)	0 (0)	
Del5q	1 (5)	0 (0)	1 (11)	
Median time since first MDS diagnosis, months (range)	17.0 (0.9–118.2)	14.4 (7.1–39.7)	25.5 (0.9–118.2)	0.713
Cytogenetic risk, n (%)				0.999
Low	17 (90)	9 (90)	8 (89)	
Intermediate	2 (10)	1 (10)	1 (11)	
High	0 (0)	0 (0)	0 (0)	
WPSS sum score, n (%)				0.211
1	2 (11)	0 (0)	2 (22)	
2	16 (85)	9 (90)	7 (78)	
3	1 (5)	1 (10)	0 (0)	
Asunercept dose, n (%)				0.628
100 mg	13 (68)	6 (60)	7 (78)	
400 mg	6 (32)	4 (70)	2 (22)	
Median CRP serum level at baseline (mg/L, range)	4.4 (2.0–25.6)	6.1 (2.0–25.6)	2.6 (2.0–11.6)	0.026
Median ANC at baseline (1/nL, range)	1.9 (0.5–5.8)	2.6 (0.5–5.8)	1.8 (1.1–3.1)	0.728
Median absolute lymphocyte count at baseline (1/nL, range)	0.9 (0.3–3.4)	0.9 (0.3–3.4)	0.8 (0.7–1.3)	0.642
Median endogenous Epo serum level at baseline (U/L, range)	157 (6–3499)	157 (6–3499)	284 (30–2147)	0.989

Abbreviations: ANC, absolute neutrophil count; ECOG, Eastern Co-operative Oncology Group; Epo, erythropoietin; CRP, C-reactive protein; IPSS, International Prognostic Scoring System; MDS, myelodysplastic syndrome. RARS, refractory anemia with ringsideroblasts; RCMD, refractory cytopenia with multilineage dysplasia; RCMD-RS, refractory cytopenia with multilineage dysplasia and ring sideroblasts; WPSS, WHO adapted Prognostic Scoring System.

**Table 2:** Median baseline serum cytokines levels [pg/mL] in responding versus non-responding patients.

	Responders (n = 10)	Non-responders (n = 9)		
	Median (IQR)	Median (IQR)	*p*	Adjusted p *
IL-18	475.5 (319.3–599.1)	804.6 (625.3–1341.9)	0.018	0.136
IFNγ	1.2 (1.1–3.8)	3.6 (2.2–8.7)	0.131	0.204
CXCL9	245.5 (95.7–368.3)	470.6 (181.4–788.0)	0.165	0.204
S100A9	97.5 (90.9–139.2)	133.2 (126.7–244.8)	0.034	0.136
IL-1β	0.1 (0.1–0.2)	1.5 (0.1–13.0)	0.167	0.204
HMGB1	4.0 (2.9–6.2)	9.0 (1.9–18.0)	0.373	0.407
IL-37	7.0 (1.1–20.2)	353.4 (3.3–1229.2)	0.155	0.204
Activin	714.3 (227.2–2086.1)	173.8 (58.6–958.8)	0.145	0.204
Follistatin	177.5 (109.3–192.5)	256.6 (165.8–371.2)	0.058	0.174
Follistatin/Activin	0.21 (0.10–0.41)	1.23 (0.14–2.80)	0.186	-
BMP7	4.5 (1.0–6.1)	2.3 (1.0–13.9)	0.750	0.750
RANKL	2.2 (0.3–6.7)	56.8 (0.3–159.5)	0.170	0.204
sP53	11.8 (1.1–13.6)	795.2 (48.2–1500)	0.024	0.136

Abbreviations: BMP7, bone morphogenetic protein 7; CXCL9, chemokine (C-X-C motif) ligand 9; HMGB1, high-mobility-group-protein B1; IL-1β, interleukin 1 beta; IL-18, interleukin 18; IL-37, interleukin 37; IFNγ, interferon gamma; IQR, interquartile range; RANKL, receptor activator of NF-κB ligand; sP53, soluble P53; S100A9, S100 calcium-binding protein A9. * According to the Benjamini-Hochberg procedure [19].

**Table 3:** Correlation matrix (Spearman rank-correlation coefficients) of baseline serum cytokines levels with each other.

	IL-18	IFNγ	CXCL9	S100A9	IL-1β	HMGB1	IL-37	Activin	Follistatin	BMP7	RANKL	sP53
IL-18	-											
IFNγ	0.514 *	-										
CXCL9	0.593 **	0.422	-									
S100A9	0.263	0.523 *	0.209	-								
IL-1β	0.256	0.453	0.666 **	0.517 *	-							
HMGB1	0.210	0.553 *	0.545 *	0.353	0.911 ***	-						
IL-37	0.273	0.471 *	0.669 **	0.516 *	0.835 ***	0.762 ***	-					
Activin	−0.531 *	−0.130	0.115	−0.391	0.279	0.339	0.284	-				
Follistatin	0.577 *	0.408	0.472 *	0.253	0.272	0.199	0.137	−0.488 *	-			
BMP7	−0.040	0.209	0.168	−0.203	−0.011	0.165	−0.092	0.293	−0.063	-		
RANKL	0.252	0.332	0.665 **	0.297	0.716 ***	0.666 **	0.894 ***	0.185	0.424	−0.173	-	
sP53	0.393	0.453	0.643 **	0.447	0.760 ***	0.672 **	0.854 ***	0.116	0.296	−0.035	0.823 ***	-

* *p* < 0.05; ** *p* < 0.01; *** *p* < 0.001. Abbreviations: BMP7, bone morphogenetic protein 7; CXCL9, chemokine (C-X-C motif) ligand 9; HMGB1, high-mobility-group-protein B1; IL-1β, interleukin-1 beta; IL-18, interleukin-18; IL-37, interleukin-37; IFNγ, interferon gamma; RANKL, receptor activator of NF-κB ligand; sP53, soluble P53; S100A9, S100 calcium-binding protein A9.

**Table 4:** Associations of baseline cytokine serum levels with non-response to asunercept treatment and corresponding predictive values.

	Odds Ratio per log2 Increase * in Baseline Cytokine		Area under the ROC Curve (AUC)	
	OR (95% CI)	*p*	AUC (95% CI)	*p*
IL-18	4.94 (1.00–24.47)	0.049	0.82 (0.63–1.00)	0.018
IFNγ	1.81 (0.85–3.88)	0.124	0.72 (0.48–0.96)	0.111
CXCL9	1.41 (0.80–2.49)	0.234	0.69 (0.44–0.94)	0.165
S100A9	7.44 (0.90–61.38)	0.060	0.79 (0.57–1.00)	0.034
IL-1β	1.87 (0.73–4.84)	0.194	0.68 (0.43–0.93)	0.191
HMGB1	1.26 (0.75–2.11)	0.391	0.63 (0.35–0.90)	0.141
IL-37	1.22 (0.95–1.57)	0.122	0.69 (0.44–0.94)	0.126
Activin	0.72 (0.45–1.15)	0.167	0.30 (0.05–0.54)	0.145
Follistatin	4.75 (0.88–25.63)	0.070	0.77 (0.53–1.00)	0.058
Follistatin/Activin	1.44 (0.88–2.35)	0.150	0.71 (0.43–0.98)	0.169
BMP7	1.12 (0.68–1.86)	0.651	0.53 (0.24–0.81)	0.860
RANKL	1.45 (0.97–2.18)	0.069	0.70 (0.45–0.96)	0.145
Soluble P53	1.31 (1.00–1.70)	0.047	0.80 (0.59–1.00)	0.027

* Each one unit increase in log2 corresponds to a doubling in the corresponding cytokine level. Abbreviations: BMP7, bone morphogenetic protein 7; CI, confidence interval; CXCL9, chemokine (C-X-C motif) ligand 9; HMGB1, high-mobility-group-protein B1; IL-1β, interleukin-1 beta; IL-18, interleukin-18; IL-37, interleukin-37; IFNγ, interferon gamma; OR, odds ratio; RANKL, receptor activator of NF-κB ligand; ROC, receiver operating characteristic; S100A9, S100 calcium-binding protein A9.
